# Member announcements

**DOI:** 10.3109/03009734.2012.661564

**Published:** 2012-02-15

**Authors:** Anna Rask-Andersen, Torbjörn Karlsson

Dear Members,

We have two forthcoming events that we would like to highlight. The first is this year’s Martin Holmdahl lecture that will take place on March 14 at 6.00 PM in the Rudbeck lecture hall. The speaker this year will be professor Göran Hedenstierna and the title of his presentation is “Varför och hur andas vi?”. This lecture is a joint project between Selanderska stiftelsen, Uppsala Medicinhistoriska förening, Uppsala Medicinska Seniorer and the Upsala Medical Society.

The traditional spring meeting of our society will take place in the month of May. This year we will visit the new “Psykiatrins hus” at the hospital premises. Under the guidance of Karin Norlén and Åsa Hagberg we will learn more about the new buildning. There will also be some presentations of activities that will be housed in the new facilities. For further details, see our web page www.upsalalakareforening.se. and separate invitations sent to all our members.

At this time of the year all members of our society have the privilege of nominating candidates for the Rudbeck prize. The name of the candidate and a short justification have to be sent to the chairman of the society no later than March 1.

